# Influence of rootstock on endogenous hormones and color change in Cabernet Sauvignon grapes

**DOI:** 10.1038/s41598-023-33089-z

**Published:** 2023-04-24

**Authors:** Zhiyu Liu, Chunmei Zhu, Junli Sun, Zhijun Zhang, Shucheng Zhao, Wenchao Shi, Wei Wang, Baolong Zhao

**Affiliations:** 1 Department of Horticulture, College of Agriculture, Shihezi University, Shihezi, 832003 China; 2 The Key Laboratory of Special Fruits and Vegetables Cultivation Physiology and Germplasm Resources Utilization of the Xinjiang Production and Construction, Xinjiang, China

**Keywords:** Biological techniques, Molecular biology, Plant sciences

## Abstract

Different rootstocks for grapes can significantly affect fruit color and quality, possibly by affecting hormone contents, related genetic pathways, and fruit coloring mechanisms in skin. ‘Cabernet Sauvignon’ was grafted to ‘5BB’, ‘SO4’, ‘140R’, ‘CS’, ‘3309M’ and ‘*Vitis riparia*’ rootstocks, with self-rooting seedlings as the control (CS/CS), and sampled from the early stage of veraison to the ripening stage. The effects of rootstock on the contents of gibberellin (GA_3_), auxin (IAA), and abscisic acid (ABA) in grape skin were determined alongside the expression levels of eight anthocyanin synthesis related genes using real-time fluorescence quantitative PCR methods. The rootstock cultivars exhibited accelerated fruit color change, and the CS/140R combination resulted in grapes with more color than the control group in the same period. With the development of fruit, the IAA and GA_3_ contents in the skin of different rootstock combinations showed trends of increasing initially, then decreasing, while the ABA content decreased initially and then increased. During the verasion (28 July), the various ‘Cabernet Sauvignon’ rootstock combinations exhibited varying degrees of increases in GA_3_, ABA, and IAA contents; correlation analysis showed that, at the start of veraison, the expression levels of the anthocyanin synthesis-related genes *VvCHS*, *VvDFR*, and *VvUFGT* had strong positive correlations with hormone contents, which indicated they are key genes involved in the endogenous hormone responsive anthocyanin biosynthesis pathway. The results of this study showed that rootstock regulates the fruit coloring process by influencing the metabolism level of peel hormones in the ‘Cabernet Sauvignon’ grape.

## Introduction

In the 1860s, to stop the spread of phylloxera in grapes, European scientists introduced American grapes as rootstocks, thus promoting the application and research of grape grafting technology^1^. Today, resistant rootstock is widely utilized for grafting because it increases yield and fruit quality while also improving the stress resistance of various grape varieties^2^. In ‘Cabernet Sauvignon’ grapes, SO4 rootstock has been found to significantly increase the anthocyanin content of fruits, enhance berry coloration, and increase tannin content^3^; Zhang et al. found that using resistant rootstock 140R improved the phenolic substances and resveratrol of skin of ‘Cabernet Sauvignon’ grapes, and also had a regulatory effect on grape fruit coloring and anthocyanin accumulation^4^. The color of the skin of wine grapes can directly affect the quality of wine and sensory quality, and is an important indicator to measure the commercial value of wine grapes^5^.

The main pigments that determine the color of RED grape berries are anthocyanins and chlorophylls. During the veraison, chlorophyll metabolism in grapes is accelerated while anthocyanins are gradually accumulated, processes which are closely controlled by hormones^6^. Plant hormones are compounds that play many roles in important metabolic pathways and, as such, can actively participate regulation of plant growth and metabolism^7^. For example, the exogenous application of abscisic acid (ABA) was found to significantly increase the anthocyanin content of ‘Isabella’ grape fruit^8^. Naphthaleneacetic acid (NAA) has been identified as a synthetic broad-spectrum plant growth regulator with similar characteristics to auxin (IAA), and He et al. found that the application of 200 mg L^−1^ NAA significantly reduced the expression of genes related to anthocyanin synthesis in grape skin^9^, indicating that exogenous NAA can inhibit the process of grape color transcoloration. Similarly, exogenous gibberellin (GA3) treatment of ‘Sangiovese’ grapes reduced the anthocyanin content of grapes^10^.

Cabernet Sauvignon is one of the most widely cultivated wine grape varieties in the world, and Xinjiang, one of the main Cabernet Sauvignon-producing regions in China, has the largest planting area of Cabernet Sauvignon. Cabernet Sauvignon is dark in color, fruity, high tannins, high acidity, aged with aromas of smoke, vanilla and coffee, and has the quality of Tibetan brewing. Understanding the effects of grafting on plant hormones and anthocyanins may be valuable in the production of Cabernet Sauvignon grapes.

Previous studies have mainly focused on the effects of grafting on the color of grapes or the effect of exogenous hormones on fruit color transfer, and few studies have examined changes in the metabolic levels of scion hormones due to grafting rootstock and how they affect the fruit color transfer process. In this study, changes in fruit color and hormones from the transcolor stage to the ripening stage of grapes with different rootstocks were monitored, as were the relative expression levels of anthocyanin synthesis related genes. These data were examined to determine the effect of grafted rootstocks on endogenous hormone contents in grape skin and their roles in the fruit color transfer process. This study enriches the influence of rootstock on the veraison of grapes, which lays a theoretical foundation and is of great significance.

## Results

### CIRG index of ‘Cabernet Sauvignon’ grapes with different rootstocks

**Figure 1:**
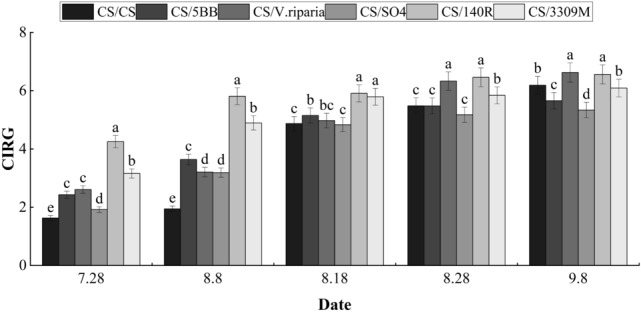
Influence of different rootstocks on the CIRG value of Cabernet Sauvignon grapes. Lowercase letters above the bars denote significant differences (P < 0.05) attested using Tukey’s HSD test. Error bars represent SE (n = 3).

The CIRG index values of grape fruits of the 6 treatments combinations are shown from the transition stage to the ripening stage in Fig. 1. From July 28 to September 8, the CIRG index values of all processed grapes showed upward trends. On July 28, the CIRG index values of the different groups were ordered: CS/140R > CS/3309M > CS/*V. riparia* > CS/5BB > CS/SO4 > CS/CS, and the CIRG index values of all treatments were an average of 176.11% higher than the control. With the growth and development of fruits, the differences in the CIRG index values between the rootstock groups became smaller and smaller, and at the ripening stage of fruit the treatments were ordered: CS/*V. riparia* > CS/140R > CS/CS > CS/3309 M > CS/5BB > CS/SO4.

### Endogenous hormone contents of ‘Cabernet Sauvignon’ grapes with different rootstocks

**Figure 2:**
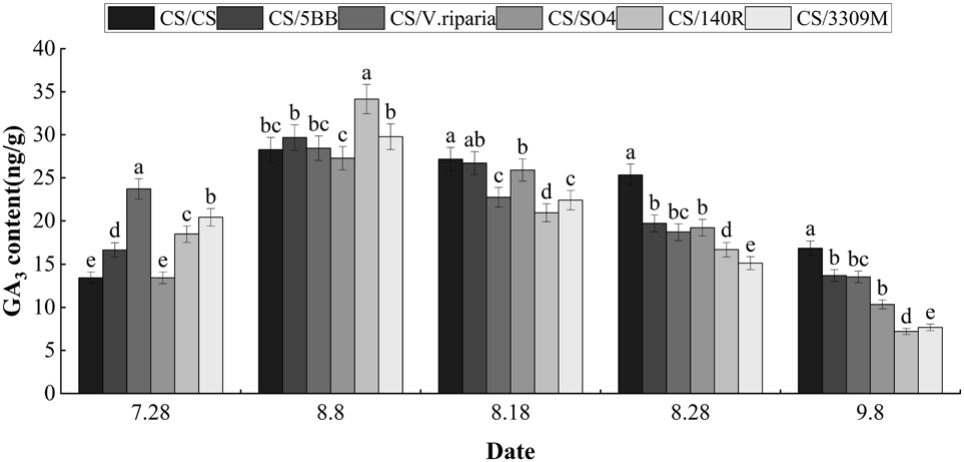
Effects of different rootstocks on GA3 content in grape skins of Cabernet Sauvignon. Lowercase letters above the bars denote significant differences (p < 0.05) attested using Tukey’s HSD test. Error bars represent SE (n = 3).

The GA3 contents of ‘Cabernet Sauvignon’ grape skin of different rootstock grafting combinations, as obtained by HPLC analysis, are shown in Fig. 2. The GA3 content in the skin initially showed increasing trends, but shifted to steadily decreasing as the berries grew and developed. The GA3 content of all treatment skin continued to increase during the grape color transition from July 28 to August 8, and the GA3 content of the treatment skin on July 28 were ordered: CS/*V. riparia* > CS/3309M > CS/140R > CS/5BB > CS/SO4 > CS/CS (control). The average GA3 contents of the treatments was 37.97% higher than that of the control, and all treatments were significant greater than the control. The GA3 content was highest in the CS/*V. riparia* peel, reaching 23.7 ng g^−1^. The control treatment had the lowest GA3 content, at 13.43 ng g^−1^. The GA3 content of the grafted combinations skin of the rootstock reached the maximum rate of increase on August 8, at which time the CS/140R treatment had the highest GA3 content, reaching 34.13 ng g^−1^. After the peak GA3 content on August 8, all treatments decreased to their lowest values on September 8, which were an average of 60.3% lower than the peak values. After this time, the GA3 content of the control treatment gradually overtook the other rootstock grafting combinations, and at the fruit ripening stage the GA3 contents of all grafted rootstock combinations were significantly lower than that of the control treatment. At ripening, the GA3 of the treatment groups were ordered: CS/CS > CS/5BB > CS/*V. riparia* > CS/SO4 > CS/3309M > CS/140R.

**Figure 3:**
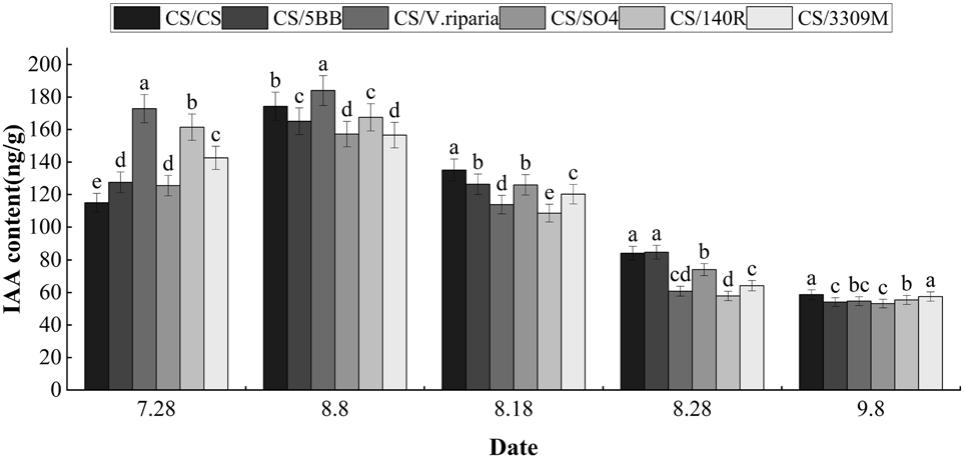
Effects of different rootstocks on IAA content in grape skins of Cabernet Sauvignon. Lowercase letters above the bars denote significant differences (p < 0.05) attested using Tukey’s HSD test. Error bars represent SE (n = 3).

As can be seen from Fig. 3, the IAA content in the grape skin during growth and development increased during the early fruit color change stage (July 28), but began to decline after August 8 until the IAA contents of the skin were reduced to their lowest levels at the ripening stage. This trend was similar to the change trend in GA3 content in the peel. The IAA content in the CS/*V. riparia* treatment during the growing season peaked at 129.3 ng g^−1^. At the start of the fruit coloration stage, the IAA contents of the skin of the different treatment groups were ordered: CS/*V. riparia* > CS/140R > CS/3309M > CS/5BB > CS/SO4 > CS/CS (control). During this period, the IAA contents of all treatments were significantly higher than that of the control, by an average of 26.95%; from August 8 to August 28, the IAA contents of all treatments decreased by an average of 66.7%, The IAA content of the control treatment was consistently at a high level among all treatments, while the IAA content of the control treatment and other treatments was not significantly different during the fruit ripening period (September 8).

**Figure 4:**
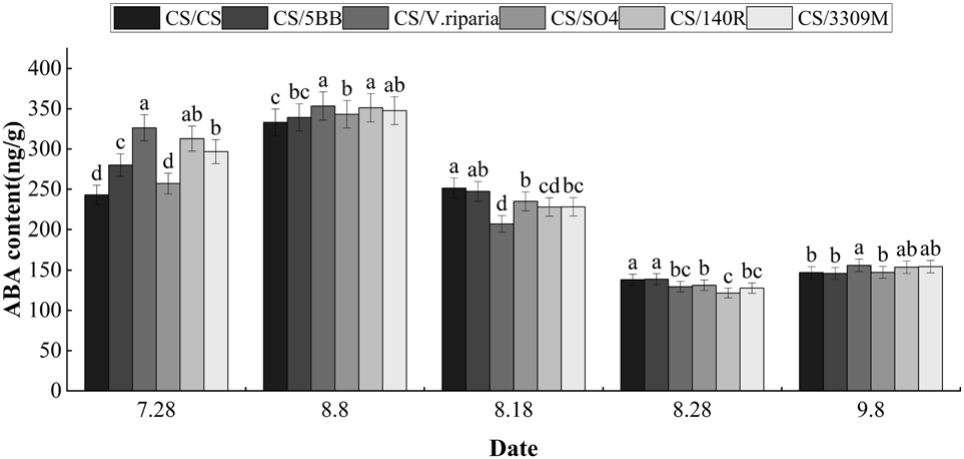
Effects of different rootstocks on ABA content in grape skins of Cabernet Sauvignon. Lowercase letters above the bars denote significant differences (p < 0.05) attested using Tukey’s HSD test. Error bars represent SE (n = 3).

During the growth and development of grape fruits, the ABA content in the skin briefly increased during the veraison, and then the ABA content of all treatments continued to decrease, and finally the ABA content in the skin increased to a certain extent during the fruit ripening stage (Fig. 4). Beginning of ripening, the ABA content of each treatment varied greatly, and the ABA contents of the groups were ordered: CS/*V. riparia* > CS/140R > CS/3309 M > CS/5BB > CS/SO4 > CS/CS (control). The average ABA content of the treatments was higher than the control by 21.26%; the ABA content of CS/V. riparia was the highest, reaching 326.2 ng g^−1^; the ABA content of the control was the lowest, at 242.87 ng g^−1^. After a brief increase in the ABA content of the peel before August 8, the ABA content of the skin of all treatments decreased from August 8 to August 28, with an average decrease of 61.9%. At the end of fruit ripening, the ABA content of the skin of each rootstock combination had slightly increased, with an average increase of 15.26% compared with the lowest level during the growth period.

### Expression of genes related to anthocyanin content and its synthesis

**Figure 5:**
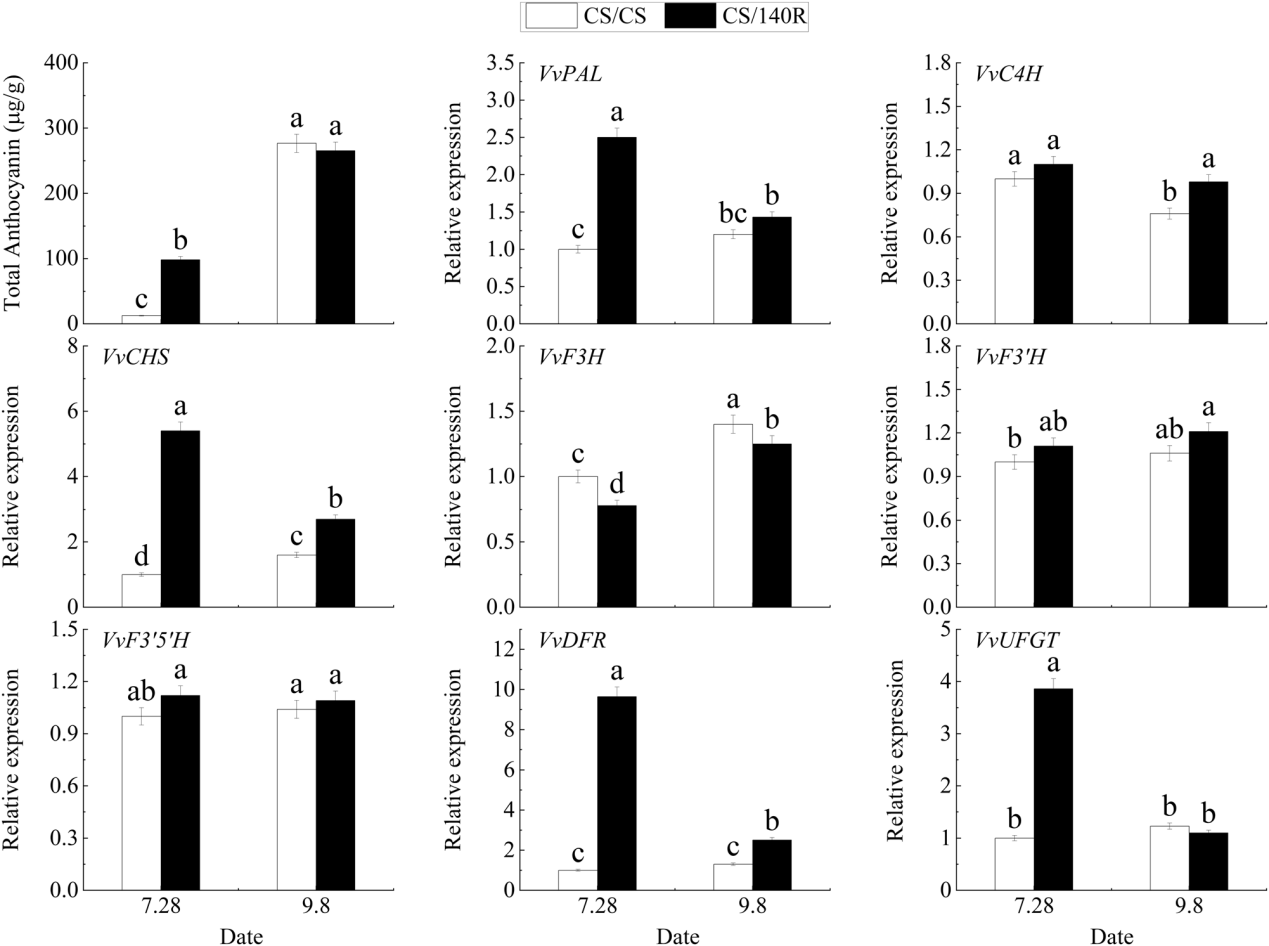
Expression analysis of anthocyanin content and genes related to its synthesis. Lowercase letters above the bars denote significant differences (p < 0.05) attested using Tukey’s HSD test. Error bars represent SE (n = 3).

To explore the mechanisms underlying the differences in anthocyanin accumulation in the skin of ‘Cabernet Sauvignon’ with different rootstocks, In the previous experiment, the combination CS/140R with the largest color change was found, and the anthocyanin contents of skin and the relative expression of the anthocyanin synthesis related genes were determined in the self-rooted grafted seedling ‘Cabernet Sauvignon’ (control) and the CS/140R rooting spike combination (Fig. 5). The analysis showed that the anthocyanin content of CS/140R during the fruit color transition period (July 28) was 7.83 times that of the control (CS/CS), but the anthocyanin contents had no significant differences during the fruit ripening period (September 8).

During the fruit color transition period, the expression levels of *VvPAL*, *VvCHS*, *VvDFR*, and *VvUFGT* in CS/140R were significantly upregulated compared to CS/CS, while the expression of *VvF3H* was down-regulated; the expression levels of other anthocyanin-synthesizing genes did not appear different. At the ripening stage, the expression levels of *VvC4H*, *VvDFR*, and *VvCHS* in CS/140R were up-regulated compared with the control, while the VvF3H gene was down-regulated; the expression levels of the other anthocyanin synthesis genes were not different between CS/140R and CS/CS.

### Correlations between grape peel hormones and anthocyanin synthesis gene expression

In this study, correlations between hormone contents and anthocyanin synthesis gene expression in the skin of fruit during the veraison was analyzed by SPSS (Table 1). The relative expression levels of the *VvCHS* and *VvUFGT* genes were significantly positively correlated with the contents of ABA and IAA (p < 0.01). VvDFR expression was significantly positively correlated with ABA content (p < 0.01) and IAA content (p < 0.05). *VvPAL* expression was significantly positively correlated with ABA and IAA content (p < 0.05), *VvF3H* was significantly negatively correlated with GA_3_ content (p < 0.05), but the expression levels of the remaining genes were not significantly correlated with any hormone contents.

**Table 1:** Correlation analysis between grape peel hormone content and anthocyanin synthesis gene expression.

Hormone	Correlation coefficient							
	*VvPAL*	*VvC4H*	*VvCHS*	*VvF3H*	*VvF3'H*	*VvF3′5'H*	*VvDFR*	*VvUFGT*
ABA	0.688*	0.462	0.928**	− 0.468	− 0.31	0.285	0.971**	0.981**
GA	0.436	− 0.05	0.338	− 0.774*	− 0.472	0.051	0.481	0.419
IAA	0.686*	0.38	0.931**	− 0.381	− 0.278	0.296	0.769*	0.919**

*Indicate significant, significant at p < 0.05.

**Indicate extremely significant effect at p < 0.01.

## Discussion

Anthocyanins, chlorophylls, and carotenoids are the three most important pigments that determine the red color of grape fruit. During the veraison, chlorophylls degrade, while anthocyanins and carotenoids gradually accumulate, changing the appearance of mature fruits depending on the proportion of the two, with the types and contents of anthocyanins determining whether the fruit is red or purple^11^. In ‘Cabernet Sauvignon’, it was found that grafting rootstock can increase the content of phenylalanine transaminase in the skin of scions from the color transfer stage to the mature stage^12^. Nam et al. found that the color of grape fruit was determined by a variety of substances^13^, and it was difficult to use chromaticity values (L*, a*, and b*) to compare fruit quality among different grape varieties during production. Furthermore, they found that the color index of red grape (CIRG) could be indexed with the anthocyanin content of grape peel, which was a reliable index to evaluate the appearance quality of grapes. Grafting ‘Cabernet Sauvignon’ to ‘M4’ and ‘1103P’ rootstock significantly improved the fruit CIRG index^14^. By measuring the fruit color of Merlot grafted on different rootstocks, it was found that ‘4453M’, ‘110R’, and ‘SO4’ rootstocks significantly increased the anthocyanin and polyphenol contents of fruit^3^. In this study, showing that the CIRG index of each rootstock grafted combination fruit in the initial fruit coloring stage was higher than the control treatment, indicating that rootstock accelerated the process of fruit coloring, which was also consistent with previous research using ‘Cabernet Sauvignon’ grapes^15^. However, with the gradual ripening of the fruit, the differences in the CIRG index values of the different treatments began to decrease, and the differences were smallest at the ripening stage when only the CS/*V. riparia* and CS/140R treatments were significantly higher than the control. This could be explained that this is because rootstocks mainly affect the coloring process of fruits, and only some rootstocks can increase the content of anthocyanins in the ripening stage of ‘Cabernet Sauvignon’ grapes.

After rootstock grafting, the growth and development of the scion is regulated by the metabolic levels of endogenous hormones, and the synthesis and transportation modes of endogenous hormones in different rootstock grafting combinations also change^16^. ABA is an important endogenous plant hormone that plays a key role in the transfer of plant signals^17^. Xu et al. found that ABA promotes the accumulation of genes related to grape anthocyanin synthesis^18^. The results of this study showed that the ABA content of each rootstock combination peaked during the color transition period before dropping to their lowest levels before the ripening stage. This trend was likely driven by the continuous accumulation of ABA in the fruit before the color change, and the ABA content of seeds being transferred to the peel during the color transition period to maintain fruit color and ripening, which is consistent with previous observations^19^. These results showed that the grafting combinations of different rootstock could significantly increase the ABA content of the peel in the early color transfer stage, indicating that the rootstock can regulate fruit color by influencing ABA content during this period.

The main physiological role of GA in grapes is to induce fruit expansion, this improves fruit set and fruit quality^20^. It has previously been found that the anthocyanin content increased and was significantly correlated to the GA content of table grapes^21^. Wang et al. found that the exogenous application of 50 mg L^−1^ GA3 affected the quality of grape fruits by elongating the fruit longitudinally and inhibiting seed development^22^. In the treatment of grape inflorescence, exogenous GA3 was found to significantly inhibit the expression of key genes involved in the synthesis of endogenous gibberellin^23^. The peak GA3 content in this study occurred in the fruit color transition stage, and the GA3 content of the peel in the early stage of color transformation was significantly elevated in the grafted groups, but the GA3 content was reduced during the fruit ripening period, indicating that rootstock grafting can affect the metabolism of endogenous GA3 in grapes, which is consistent with the conclusion of Wang et al.^16^.

During grape growth and development, IAA has been observed to promote fruit ripening and participate in the regulation of other hormone metabolism levels^24^. Corso et al. compared the changes in IAA in the skin and pulp of grapes after grafting to two kinds of rootstock and found that rootstock significantly altered the metabolic level of IAA^14^, which was particularly evident in the peel. These studies also found that the content of IAA decreased from the beginning of the grape fruit color transition period, and that IAA inhibited the ripening of grapes during this time^25^. In this study, the IAA contents in the skin of each Rootstock-scion combination continued to decrease after their peaks in the color transition period, and the IAA content in the skin of the control after the color transition gradually became higher than the other treatments, indicating that rootstock grafting promoted fruit ripening by reducing the IAA content after the color transition.

The biosynthesis of anthocyanins in grape skin is mainly carried out by the flavonoid pathway, a process controlled by structural genes that regulate the formation of multi-enzyme complexes by various structural enzymes downstream, which in turn catalyze anthocyanin biosynthesis^26^. One study observed a significant correlation between anthocyanin synthesis structural genes and plant color changes^27^. In ‘Summer Black’ grapes, it was found that increases in anthocyanin levels were mainly due to the upregulation of structural genes such as *PAL*, *C4H*, *4CL*, *CHS*, *DFR*, and *ANS*^28^. In this experiment, the anthocyanin content in grape skin was determined at the veraison, and qRT-PCR analysis was performed to measure the expression levels of *PAL*, *C4H*, *4CL*, *CHS*, *F3*′*H*, *F3*′*5*′*H*, *F3H*, *DFR*, and other structural genes. The anthocyanin content in CS/140R treatment was much higher than that in the control, this corresponded to the up-regulation of the *VvCHS*, *VvDFR*, *VvPAL*, and *VvUFGT* genes, indicating that the rootstock played an important role in regulating the fruit color transformation.

Plant hormone levels are not static while regulating fruit coloring, they have different roles at different times. Gao et al. found that the regulatory role of the ABA hormone on other hormones changed during different periods of fruit coloring^19^, exhibiting a significant positive correlation with GA3 content in the early stage color transfer, which promoted fruit coloring, but a significant positive correlation with IAA and ethylene content during fruit ripening, possibly related to promoting fruit ripening. Treating the fruit with exogenous ABA significantly up-regulated the expression of *CHI*, *F3H*, *DFR*, and *UFGT*, as well as the *VvMYBA1* and *VvMYBA2* transcription factors in different grape varieties^29^. At present, the molecular mechanisms by which fruit hormones interact to regulate anthocyanin synthesis are poorly understood, so this study used correlation analysis to reveal the relationships between hormones and structural gene expression in grape skin, finding that ABA and IAA were positively correlated with structural the genes *VvCHS*, *VvDFR*, and *VvUFGT*. It was therefore speculated that *VvCHS*, *VvDFR*, and *VvUFGT* are key to the hormone-responsive anthocyanin synthesis pathway, and that ABA and IAA have synergistic roles in regulating anthocyanin synthesis, but these conclusions require further experimental verification.

## Conclusions

In summary, rootstock had a great influence on the hormone content and coloration of grafted ‘Cabernet Sauvignon’ grape skin. Rootstocks affected the fruit coloration process by influencing hormone metabolism levels in scion fruits. The qRT-PCR analysis of anthocyanin synthesis genes showed that certain structural genes were involved in regulating anthocyanin synthesis, and were significantly positively correlated specific endogenous hormones. Therefore, these may be key genes in the hormone responsive anthocyanin synthesis pathway. However, in this experiment, only the total content of anthocyanins was measured and different types of anthocyanins were not examined, so the specific influences of rootstocks on different types of anthocyanins will need to be determined in future research.

## Materials and methods

### Materials and handling

The experimental vineyard was located in the College of Agronomy of Shihezi University in Xinjiang, Vines are grown in sandy soil at 2 × 3 m under drip irrigation system, and all experimental materials are obtained in this experimental garden. The resistant rootstocks for the test were ‘5BB’, ‘SO4’, ‘140R’, ‘3309M’, and ‘*Vitis riparia*’ from the Zhengzhou Institute of Pomology of the Chinese Academy of Agricultural Sciences, the rootstocks used and their characteristics were shown in the table below (Table 2)^30^, and the scion material was ‘Cabernet Sauvignon’ (CS) in the wine region (Xinjiang, China). A total of 6 treatments were set up in the experiment, specifically CS/5BB, CS/SO4, CS/140R, CS/3309M, CS/*V. riparia*. Ten vines were planted per treatment, self-rooted grafted seedlings (CS/CS, control) were colonized in the grape test garden in 2015, with a plant row spacing of 1 m × 3 m, using the hedge cultivation mode with low factory glyph and V-shaped leaf curtain.

**Table 2:** Characteristics of main rootstock varieties.

Variety type	Country	Main characteristics
*V. riparia*	French	It is extremely resistant to phylloxera, but the leaves often carry worms. It is easy to take root, easy to graft, and small feet are prone to appear after grafting, but it does not affect the life of the grapes
SO4	Germany	The growth is vigorous and the initial growth is very rapid. Easy fruit set and early ripening. Suitable for wet clay, not drought resistant, strong salt resistance. Easy to root, good for reproduction. The grafting is in good condition
5BB	Austria	The growth is vigorous, the yield is large, the rooting is good, and it is conducive to reproduction. Suitable for moist, cohesive soils. Not suitable for extreme drought conditions. Resistant to nematodes
140R	Italy	The growth is extremely vigorous and the resistance to calcareous soils is excellent. Root system against phylloxera. Cuttings are more difficult to root and easy to graft in the field
3309M	French	The plant grows strongly. It has strong resistance to phylloxera, drought resistance and root cancer resistance

The trial began on July 28, 2020 (fruit transition phase), berry sampling took place every 10 days starting at veraison until full ripening. Samples of three berries per vine were collected randomly from ten vines for each combination (30 berries per sample). As a result, five samples were collected until harvest. Each sampling process was conducted randomly and repeated 3 times, and brought back to the laboratory immediately after sampling. The peel was carefully separated from the pulp and seeds and frozen it in liquid nitrogen before being place it in a − 80 °C freezer until the determination of hormones. The treated group that exhibited the greatest difference in color from the control (CS/CS) during the fruit transition phase was selected for anthocyanins assay and RNA extraction.

### Determination of physiological indicators

A portable chromatic aberration spectrophotometer (TianJin Toolso Technology Co. Ltd.) was used to determine the L, a, and b values of the skin chromatic aberration, which were used to calculate the C value, h value, and CIRG index, that is, C (color saturation, Chroma) = (a2 + b2) 1/2, h (hue value, hue) = arctangent b/a, CIRG (red grape fruit color index, color index of red grape) = (180 − h)/(L + C). The endogenous plant hormones were determined using a slightly modified high performance liquid chromatography method: 0.5 g of plant material was ground into powder while frozen with liquid nitrogen and 10 mL of pre-chilled 80% methanol solution was added before placing the mixture in a 4 °C refrigerator for 10 h; the mixture was then centrifuged at 10,000 r for 15 min at 4 °C, and 5 mL of the supernatant was extracted twice, waiting 1 h between extractions. The extracted supernatants were combined and then evaporated to 1/3 of the original volume in a reduced pressure rotary steam analyzer. A volume of petroleum ether equivalent to the evaporated portion was added for decolorization and the ether phase was discarded, this was repeated three times. Using 0.1 mol L^−1^ HCl solution, the filtrate pH was adjusted to 3, then an equal volume of ethyl acetate solution was added and the combination was vortexed for 3 min; the ester phase was removed and the process was repeated three times. The combined ester phases were evacuated under reduced pressure, then dissolved in chromatographic grade methanol to a volume of 1 mL and the solution was measured using a Shimadzu LC-2010AHT type high performance liquid chromatography^31^.

Grape fruit skin anthocyanin content determination was performed by pH difference method. 1 g skin (3 replicates) was extracted with 1% HCl methanol and the absorbance was determined at 530 and 657 nm. The formula *A* = *A*530 − 0.25 *A*657 was used to calculate the contribution of chlorophyll and its degradation products to the absorbance at 530 nm. The anthocyanin concentration was a relative value, and the calibration were set as *A* = 0.01 equal to 1 unit^32^.

### Determination of gene expression

The total RNA extraction in this experiment was conducted using an RNA extraction kit (Meiji Biotechnology Co., Ltd.), and RNA quality was identified by micro-ultraviolet spectrophotometer and agarose gel electrophoresis. Synthesis of cDNA strands was achieved via reverse transcription using the RT-5-UHUUK Reverse Transcription Kit (Shanghai Yisheng Biotechnology Co., Ltd.) and stored at − 20 °C.

Primer Premier 6.0 was used to design quantitative detection primers for the eight anthocyanin-synthesis-related genes (*VvPAL*, *VvC4H*, *VvCHS*, *Vv4CL*, *VvF3H*, *VvF3’H*, *VvF3′5*′*H*, and *VvDFR*)^33^(Table 3). Taking the Actin1 gene as the internal reference gene, the RT-GGG kit (Shanghai Yisheng Biotechnology Co., Ltd.) was used for qRT-PCR reactions. The total volume of the amplification system was 20 μL: template cDNA 2 μL, SYBR Mix 10 μL, ddH_2_O 7.2 μL, forward primer and reverse primer 0.4 μL each. The amplification procedure was: 95 °C prevariation for 5 min, 95 °C denaturation 10 s, 60 °C annealing 30 s, 72 °C extension 10 s; 35 cycles. The relative expressions of genes were calculated using the 2^−ΔΔCt^ method^34^.

**Table 3:** Primer sequence of anthocyanin synthesis gene.

Gene	Forward primer	Reverse primer
*VvPAL*	CTCCTCCCCTCCATTTTCCCTTAC	TGTGTGGCAGTGGTTTCCATTTG
*VvC4H*	AAAGGGTGGGCAGTTGAGTT	GGGGGGTGAAAGGAAGATAT
*VvCHS*	GTTCTGTTTGGATTTGGACCAG	GTGAGTCGATTGTGTAGCAAGG
*VvF3H*	GCAGACTGTCCATAGCAACATTCC	CACTGCCTTCTCTCCCTCTCTTATC
*VvF3'H*	CAACAAGAGCTGGACGCAGT	AGCCGTTGATCTCACAGCTC
*VvF3′5*′*H*	AAACCGCTCAGACCAAACC	ACTAAGCCACAGGAAACTAA
*VvDFR*	GGCCAAATCAAACTACCAGA	GAAACCTGTAGATGGCAGGA
*VvUFGT*	GGGATGGTAATGGCTGTGG	ACATGGGTGGAGAGTGAGTT
*actin1*	CTTGCATCCCTCAGCACCTT	TCCTGTGGACAATGGATGGA

### Data analysis

Analysis of variance (ANOVA) and correlation analysis were used to examine the data for significant differences and relationships using SPSS 25.0, with a significance level of p < 0.05. Excel 2010 and Origin2021 were used to collate and map the data.

### Guidelines

All local, national or international guidelines and legislation were followed in the current study. The use of plant parts in the present study complies with international, national and or/institutional guidelines.

## Supplementary Information

Supplementary Information.

## Data Availability

All data generated or analysed during this study are included in this published article (and its Supplementary Information files).
